# Characterization of Aerosols of Titanium Dioxide Nanoparticles Following Three Generation Methods Using an Optimized Aerosolization System Designed for Experimental Inhalation Studies

**DOI:** 10.3390/toxics5030014

**Published:** 2017-07-01

**Authors:** Igor Pujalté, Alessandra Serventi, Alexandra Noël, Denis Dieme, Sami Haddad, Michèle Bouchard

**Affiliations:** 1 Department of Environmental and Occupational Health, Chair in Toxicological Risk Assessment and Management, and University of Montreal Public Health, Research Institute (IRSPUM), University of Montreal, Roger-Gaudry Building, U424, P.O. Box 6128, Main Station, Montreal, QC H3C 3J7, Canada; igor.pujalte@umontreal.ca (I.P.); denis.dieme@umontreal.ca (D.D.); sami.haddad@umontreal.ca (S.H.); 2 Institute of Research of Hydro-Quebec (IREQ), 1800, boul. Lionel-Boulet, Varennes, QC J3X 1S1, Canada; serventi.alessandra-maria@ireq.ca; 3 Department of Comparative Biomedical Sciences, School of Veterinary Medicine, Louisiana State University, Baton Rouge, LA 70803, USA; lexy_noel@yahoo.com

**Keywords:** nanoparticles, TiO_2_, aerosol generators, collision jet, dust jet, electrospray aerosolizer

## Abstract

Nanoparticles (NPs) can be released in the air in work settings, but various factors influence the exposure of workers. Controlled inhalation experiments can thus be conducted in an attempt to reproduce real-life exposure conditions and assess inhalation toxicology. Methods exist to generate aerosols, but it remains difficult to obtain nano-sized and stable aerosols suitable for inhalation experiments. The goal of this work was to characterize aerosols of titanium dioxide (TiO_2_) NPs, generated using a novel inhalation system equipped with three types of generators—a wet collision jet nebulizer, a dry dust jet and an electrospray aerosolizer—with the aim of producing stable aerosols with a nano-diameter average (<100 nm) and monodispersed distribution for future rodent exposures and toxicological studies. Results showed the ability of the three generation systems to provide good and stable dispersions of NPs, applicable for acute (continuous up to 8 h) and repeated (21-day) exposures. In all cases, the generated aerosols were composed mainly of small aggregates/agglomerates (average diameter <100 nm) with the electrospray producing the finest (average diameter of 70–75 mm) and least concentrated aerosols (between 0.150 and 2.5 mg/m^3^). The dust jet was able to produce concentrations varying from 1.5 to 150 mg/m^3^, and hence, the most highly concentrated aerosols. The nebulizer collision jet aerosolizer was the most versatile generator, producing both low (0.5 mg/m^3^) and relatively high concentrations (30 mg/m^3^). The three optimized generators appeared suited for possible toxicological studies of inhaled NPs.

## 1. Introduction

Due to their nanosize, nanoparticles (NPs) have unique physical and chemical properties, making their use attractive to several industries. They have already been introduced into a number of industrial processes and products. Consequently, exposure to NPs can occur during synthesis, use and disposal of products containing embedded manufactured NPs [1]. Workers are at risk of exposure, especially through inhalation. Indeed, NPs can easily be released in the air in an aerosol form, and inhalation is generally considered as the primary route of occupational exposure to NPs. Once inhaled, NPs may reach the deep lung and alveolus; this region is the primary site of deposition for particle size under 100 nmp [2]. While there are currently no published epidemiological studies of workers’ exposure to manufactured NPs, and few studies related to human exposure, a growing number of studies are conducted on animals using respiratory tract exposure to determine the toxicokinetics and toxicity of NPs, for example, Noël et al. [3]. Intratracheal instillation and inhalation are two common administration methods to expose animals. NP instillation is the most widely used method; however, it does not reflect real exposure conditions (less uniform particle deposition and nonhomogeneous distribution) [4,5]. It can also induce strong biological responses (inflammation, oxidative stress) in comparison to an exposure by inhalation [6].

Inhalation exposure is more precise than instillation and reflects realistic exposure [7]. It can be done by aerosolizing NP powders or corresponding suspensions. Several types of aerosol generators exist; the most used are powder-based generators, which disperse NPs from dry powder, and nebulizer or atomizer-type generators, which disperse NPs from a solution or suspension [8]. The choice of the NP generation method essentially depends on the aerosol characteristics sought for the experimentation. To date, a majority of studies have performed inhalation studies providing large aggregated/agglomerated NPs (>100 nm) due to technical limitations [9]. Indeed, the small size of NPs leads to their aggregation/agglomeration due to their dynamic motion in the aerosol and their high surface reactivity [1,8]. For instance, Oyabu et al. [10] reported the generation of nickel oxide (NiO) aerosols by nebulization; NP aggregates/agglomerates reached a geometric mean of 139 nm for generations with NPs of 20 nm of initial particle diameter. Ravenzwaay et al. [11] reported titanium dioxide (TiO_2_) NP aggregates/agglomerates of 200 nm following generations by nebulization, with NPs of 88 nm of initial particle diameter. More recently, Lindberg et al. [12] reported the generation of TiO_2_ aerosols with an initial particle diameter of 21 nm and an aggregated/agglomerated diameter in the aerosol of 144 nm. While there are many techniques and methods, it is difficult to generate aerosols with a nanosize distribution. Recently, an electrospray aerosolizer method was developed to generate fine aerosols from suspension of Au and NiO NPs [13,14,15]. Conventional generation methods need to be adapted to produce finer and more stable aerosols by using, for example, sedimentation compartments to select only the largest particles or pressurized airflow to fragment the larger agglomerated particles in the generator or in the aerosol [16]. Moreover, in some studies, NP aerosols were generated in partially controlled conditions; this produces NP characteristics that can differ substantially from the starting nano-powder. In order to ensure having valid research methodologies in nanotoxicology, it is necessary to generate stable aerosols that are uniform and reproducible [17]. The methods used to reach the targeted concentrations, and the particle size distribution of the aerosol, must be flexible to suit the conditions of exposure, and be stable throughout the generation period.

The goal of this work was to perform inhalation exposure experiments with a novel rat inhalation device, to characterize aerosols of TiO_2_ NPs generated, using three types of generators (i.e., a wet nebulizer aerosolizer, a dry dust aerosolizer and an electrospray aerosolizer). TiO_2_ nanopowders were selected to test aerosols, given that they are among the most produced and used NPs in the world [18]. Their wide use allows for the opportunity to compare results with those of other studies. They were also selected in our study because of their different physical characteristics that may affect aerosolization [17]. The aerosol should have the following characteristics: (1) particles that are monodispersed, with preferably small geometric diameter (<100 nm) and small geometric standard deviation (*σ_g_* < 2); (2) realistic mass concentrations that are applicable for animal experiments representative of real-life exposure conditions; (3) stability for continuous daily (≤8 h) or repeated exposure over several days (up to 21 days). For each of the three generators, the various settings and parameters influencing the aerosol characteristics were evaluated. Finally, generators were compared to each other, in order to identify the type of aerosol successfully displaying the characteristics required for an optimal inhalation exposure study.

## 2. Materials and Methods

### 2.1. Nanoparticles

TiO_2_ NPs of 7 and 50 nm were purchased from MK Impex Corp. (Mississauga, ON, Canada). The manufacturer specifications indicated that the powdered material was composed of TiO_2_ anatase (95% for 7 nm and 98% for 50 nm), with an average primary particle size of 7 nm and 50 nm. TiO_2_ of 20 nm were purchased from Nanostructured & Amorphous Materials Inc. (Houston, TX, USA). The manufacturer specifications indicated that the powdered material was composed of TiO_2_ anatase (99%), with an average primary particle size between 10 and 30 nm and a specific surface area of 200–220 m^2^/g.

### 2.2. Aerosol Generation

**Figure 1:**
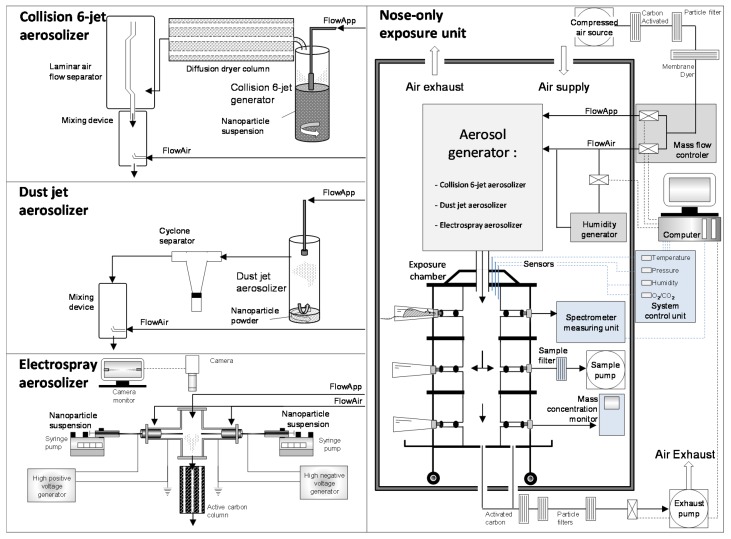
Experimental setup for aerosol generation and characterization.

In this study, a wet and a dry dust aerosolizer and an electrospray aerosolizer have been adapted and used to produce nanoaerosols. The first aerosol generator tested was a collision 6-jet wet aerosolizer (BGI Inc., Waltham, MA, USA) that can eject a suspension in a high velocity air stream to form liquid aerosols [8] (Figure 1). The NP suspension was prepared from NP powder, in ultrapure deionized water (18 mΩ) and used at a concentration between 0.5 and 10 mg/mL. A constant stirring was applied to a 125 mL of aqueous suspension of NPs (120 rpm). This suspension was sprayed with compressed air and droplets impacted against the inside of the glass vessel (370 cm^3^) to remove the larger fractions of droplets and generate an aerosol. The larger aerosol droplets were returned to the liquid stock suspension. The generated aerosol was carried out to a nebulizer and the fine aerosol fraction passed through a diffusion dryer column composed of silica beads to remove water vapor. A laminar airflow separator discarded of the larger aerosolized particles. Before entering the exposure unit, the aerosol was turbulently mixed with the incoming compressed air allowing the fractionation of the larger particles. The generator can be operated with fixed conditions according to manufacturer specifications. Generator airflow rates operate between 3.5 L/min and 20 L/min, with a maximum pressure of 2.76 bars in a 1.5 L cylinder; dilution airflow rate operates at 20 L/min, with incoming compressed air in a 0.5 L cylinder.

The second aerosol generator tested was a dust jet aerosolizer (TSE Systems GmbH, Hochtaunuskreis, HE, Germany) that provided a pneumatic dispersion of dry dust [8] (Figure 1). Dry NP powders were filled at the bottom of a flask and stirred to prevent compaction of the powder (15 cm^3^ of powder; stirring at 120 rpm). Compressed air was driven through a nozzle head with three small jets. The high velocity jets of air disturb and separate particles from the powder surface to form an aerosol. The generated aerosol was carried out to the generator and a cyclone separator separated out the larger particles. Before entering the exposure unit, the aerosol was turbulently mixed with incoming compressed air, allowing the fractionation of the larger particles. According to manufacturer specifications, the generator was operated with a purified airflow rate between 0.7 and 5 L/min, and a maximum pressure of 6 bars in a 250 cm^3^ vessel; the distance between the powder and jet nozzle was efficient at 13 cm. The dilution airflow rate was set at 20 L/min, with incoming compressed air in a 0.3 L cylinder.

The third generator tested was an electrospray aerosolizer (TSE Systems GmbH, Hochtaunuskreis, HE, Germany), which consists of applying a high voltage to a liquid suspension that is flowing through capillaries (Figure 1). The liquid jet is broken down in small droplets by Coulombic repulsion between highly charged electrodes [16,19]. A stable and concentrated suspension of NPs (between 100 and 300 mg/mL) was prepared in a 50% (*w*/*w*) solution of ultrapure deionized water (18 mΩ) and 2-propanol (HPLC grade, 200-611-7, Sigma-Aldrich, St Louis, MS, USA) allowing the control of the conductivity of the NP suspension (170 μS/cm). This suspension was placed in two syringes of 50 mL. According to manufacturer specifications, two syringe pumps (Model 540060-HP, TSE Systems GmbH, Hochtaunuskreis, HE, Germany) were set to feed the electrospray with the suspension at flow rates between 5 and 100 μL/min through two metallic capillaries (170 mm length, 1 mm I.D., 1.2 mm O.D.) separated by a distance of 3 cm. A high positive and high negative voltage was generated and applied to each metal capillary. The voltage can be changed from −10 to 10 kV and depends on the electrospray jet shape, which can be visually monitored by a CCD camera. The electrospray operating in the cone–jet mode occurs by the balance of divergent electric fields and the surface tension of the droplet at the tip of the capillary, resulting in a liquid cone with a tiny jet emitted from the tip. It is important to have two similar cone outlet capillaries to ensure that the residual charge of the aerosol is neither positive nor negative. In the present system, having two inputs with two pumps and two capillaries, one positive and the other negative, neutralized the droplet charges. Before entering the exposure unit, the generated aerosol was mixed with incoming compressed air (flow rate of 10 L/min), and passed through an active carbon column to efficiently remove the 2-propanol vapors by diffusion and absorption onto activated carbon pellets.

### 2.3. Inhalation System and Aerosol Characterization

An air compressor was used as a source of compressed air. Air was cleaned and dried by passing through cartridges with activated charcoal and through air dryer membranes. An oil mist filter and a high performance particle filter removed particles from compressed air stream. The air stream then split into two lines, the first one (Flow application) allowed the generation in the nebulizer, while the other stream (Flow air) allowed the dilution of the generated aerosol (Figure 1). The flow rate of air in both lines was regulated and controlled by a mass flow controller (TSE Systems GmbH, Hochtaunuskreis, HE, Germany). After being generated, aerosols were directed to an exposure chamber, with an internal volume of 4.9 L. The inhalation system allows for a uniform exposure of 30 rats simultaneously. The unique presentation of the nose of rats to the aerosol allows major exposure of NPs by the respiratory route, and minimizes the other routes of exposure (ingestion, ocular or dermal absorption) [20]. The temperature, relative humidity, internal pressure, O_2_ and CO_2_ rates were followed online in the chamber, and were optimized for future exposures with animals. Thus, it was necessary to have sufficient airflow (0.5 L/min/rat), an optimal temperature of 22 °C, O_2_ levels greater than 19% and CO_2_ levels lower than 0.2%. Exhaust air was connected and filtered (particles and carbon filter) by a vacuum pump controlled by the mass flow controller; this facilitated having a constant pressure in the exposure chamber (± −1 mbar). All experiments were conducted in a safety ventilated glass cover (1.79 m^3^), protecting the personnel against direct exposure to the test aerosols. This ventilated glass cover was constantly kept in negative pressure conditions to ensure minimal exposure outside the chamber.

The time-integrated mass concentration of the aerosols generated in the exposure chamber was measured by gravimetric analysis of a borosilicate glass filter of 50 mm (700-800-FI, TSE Systems GmbH, Hochtaunuskreis, HE, Germany) sampled at the flow rate of 2 L/min. The filter was weighed pre- and post-generation with a calibrated micro balance (XP2U, Mettler-Toledo Inc., Tampa, FL, USA). Online estimation of mass concentration was followed and adjusted using a DustTrak Aerosol Monitor (Model 8520, TSI Inc., Shoreview, MN, USA). The mass concentration was recorded online with a DustTrak aerosol monitor every 10 seconds and means, min and max were reported for all the generation times. The size distribution of the aerosol in the exposure chamber was monitored and measured online with a nanospectrometer (NanoSpectroPan, TSE Systems GmbH, Hochtaunuskreis, HE, Germany). This device was equipped with 2 measurement units; (1) an optical aerosol measuring unit that computes particles in the optical equivalence diameter range of 0.250 to 35.2 μm; and (2) a nanoparticle measuring unit using an electrical sensor for particle assessment in the electric mobility range of 10 to 193 nm. This device requires 1 minute per scan at a sample flow rate of 1.2 L/min. To describe the distribution of particles in the aerosol, geometric means, *d_g_*, and geometric standard deviation, *σ_g_* were calculated with the following equations [8]:

$$\ln\;d_{g}=\frac{\sum^{\text{​}}n_{i}\ln\;d_{i}}{N}\;\ln\;\sigma_{g}={\left(\frac{\sum^{\text{​}}n_{i}\;{\left(\ln\;d_{i}-\ln\;d_{g}\right)}^{2}}{N-1}\right)}^{0.5}$$

where *N* is the total number of particles (particles/cm^3^), and for each channel *_i_*, *d_i_* is the midpoint diameter (nm) and *n_i_* the number of particles (particles/cm^3^).

### 2.4. Generation Characteristics Assessed

The three aerosol generation methods (collision jet, dust jet and electrospray) were first assessed for their ability to produce stable and reproducible aerosols through time, applicable for animal inhalation exposure, with the following characteristics: small geometric diameter (<100 nm), small geometric standard deviation (<2), realistic mass concentrations (<50 mg/m^3^), low variation in the number of particles (<10%). Aerosols were thus generated at different concentrations with 20 nm TiO_2_ NPs. When aerosols were stable, their characteristics were followed online for a minimum duration of 1 h, and the size distribution and mass concentration were recorded. The long-term stability of the aerosol was assessed by recording variations in mass concentration and by following the stability of particle numbers over a minimum of 8 h, at 3 different concentration levels (low, mid and high concentrations), within the possible limits of each generator. Repeated exposure was evaluated over 21 days, with the same generation conditions for a minimum generation time of 1 h per day.

Secondly, exposure conditions affecting the aerosol characteristics were assessed. This was achieved by varying the settings and parameters of generation that could influence the aerosol characteristics, and by recording the stability of particle numbers and size distribution over 1 h. For the collision jet aerosolizer, effects of variations in air flow rates and the suspension concentrations were assessed; other conditions, such as the sonication of the suspension, the use of deionized water and the effects of using a laminar airflow separator were also tested. For the dust jet, effects of variations in the air flow rates and the use of a cyclone airflow separator were evaluated. For the electrospray, effects of variations in the suspension rate were evaluated. The stability of particle numbers and mass concentrations were recorded over 1 h. Finally, the influence of the initial NP size was assessed by generating aerosols with different NP initial size (5, 20 and 50 nm) and recording the number of particles and mass concentration for 1 h.

### 2.5. Characterization of TiO_2_ Powders and Particles in the Aerosols

TiO_2_ powders used for the generation of NP aerosols were also characterized by Transmission Electron Microscopy (TEM), X-Ray Diffraction (DeVinci, Bruker, Billerica, MA, USA) and Raman Spectroscopy (LabRam, Horiba, Kyoto, Japan). For the characterization of aerosols generated in the inhalation chamber with the dust jet and collision jet generators, TiO_2_ particles were collected on amorphous carbon holey films mounted on Cu grids and examined by an E-TEM (HF 3300, Hitachi, Tokyo, Japan) operated at 300 kV. This microscope facilitates observation of a large variety of materials under vacuum conditions or in a gas atmosphere. It is equipped with conventional and high resolution imaging modes, electron diffraction modes (selected area electron diffraction and nano-diffraction mode); electron dispersive X-ray spectroscopy (EDX of Gatan, Pleasanton, CA, USA) and electron energy loss spectroscopy (EELS of Gatan, Pleasanton, CA, USA). This analysis was performed at the Material Characterization Laboratory of the Institute of Hydro-Québec (Varennes, QC, Canada).

## 3. Results

### 3.1. Assessment of Aerosol Generation Method

**Figure 2:**
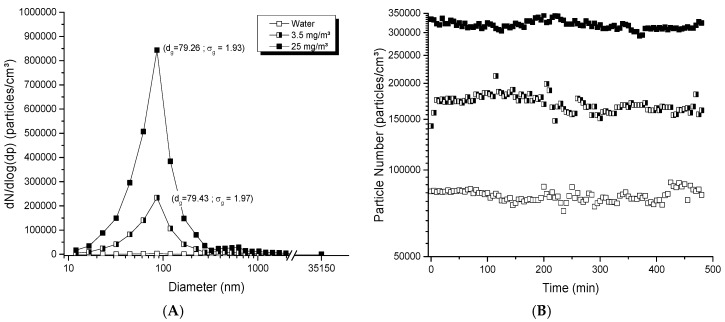

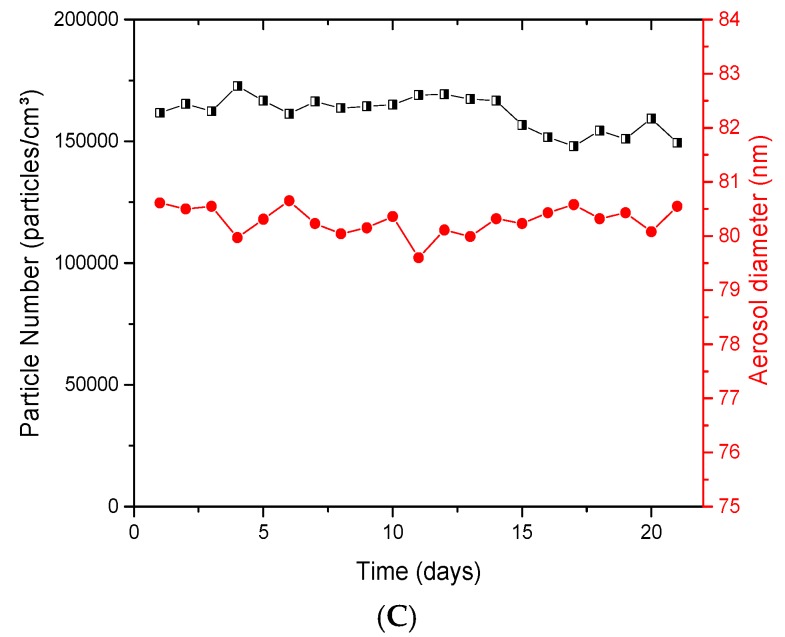
Evaluation of aerosols generated with the collision jet generator: (**A**) size distribution of typical titanium dioxide (TiO_2_) aerosols as function of mass concentration; (**B**) stability of aerosols during 8 h; (**C**) long-term stability of the number of particles and diameter in the aerosols over 21 days. The TiO_2_ (20 nm) suspension concentrations used were: in (**A**), 0, 1 and 10 mg/mL with an airflow rate of 5 L/min; in (**B**), 5 mg/mL with 3.5, 5 and 7 L/min; in (**C**), 5 mg/mL with 5 L/min to generate an aerosol per day during 21 days. The red points represent diameters of particles in the aerosol (nm) monitored at specific time points over 21 days (see right ordinal axis).

The collision jet generator was able to generate high and low concentrations, from 0.5 to 30 mg/m^3^. Aerosols displayed a homogeneous and unimodal size distribution (Figure 2A), with geometric mean diameters below 80 nm and small geometric standard deviations (*σ_g_* <2). Figure 2B shows the stability of aerosol particle number over 8 h; for respective averages of 81,300, 170,000 and 318,000 particles/cm^3^, low variations of 4.53, 6.62 and 4.20% were observed. Figure 2C depicts the stability in the average number of particles and geometric mean diameters evaluated daily over 21 days. Stable number of particles ranging between 148,000 and 173,000 particles/cm^3^ and stable particle diameters of 79.6 to 80.6 nm were observed over time (Figure 2C). All of these results showed the ability of this generator to provide a good dispersion of NPs, and stable generation applicable for daily and day-to-day exposures.

**Figure 3:**
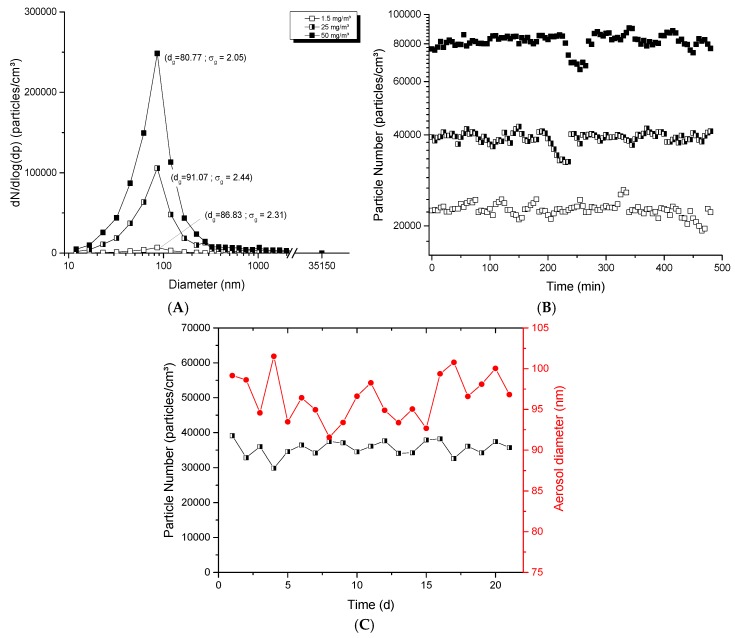
Evaluation of aerosols generated with the dust jet generator: (**A**) size distribution of typical TiO_2_ aerosols as function of mass concentration; (**B**) stability of aerosols during 8 h; (**C**) long-term stability of the number of particles and diameter in the aerosols over 21 days. The TiO_2_ (20 nm) nanopowders were used with an airflow rate of 0.7, 1 and 4.5 L/min in (**A**); 0.8, 0.9 and 1.25 L/min in (**B**); 0.9 L/min in (**C**) to generate an aerosol per day during 21 days. The red points represent diameters of particles in the aerosol (nm) monitored at specific time points over 21 days (see right ordinal axis).

With the dust-jet generator, the mass concentrations generated in the exposure chamber were higher compared to the two other generators; the concentrations could vary from 1.5 to 150 mg/m^3^. Figure 3A presents typical aerosol products; aerosols showed homogeneous profiles with unimodal distribution and produced aggregates/agglomerates displaying geometric mean diameters between 80 and 100 nm. Figure 3B shows the stability of aerosols over 8 h using 20 nm nanopowders at airflow rates of 0.8, 0.9 and 1.25 L/min to generate low, mid and high aerosol concentrations. Variations of 4.95, 4.78 and 5.69% over 8 h were observed for respective averages of 22,600, 39,000 and 81,500 particles/cm^3^. The repeated stability over 21 days is presented in Figure 3C. Stable average (± SD) number of particles of 35,500 ± 2200 particles/cm^3^ and stable particle diameters of 96.5 ± 2.8 nm were obtained. These results show the ability of the dust-jet generator to produce nanoaerosols with high mass concentrations applicable for single day and repeated day-to-day exposures.

**Figure 4:**
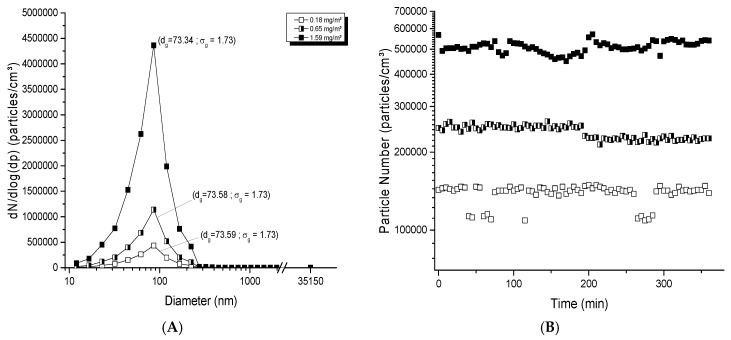

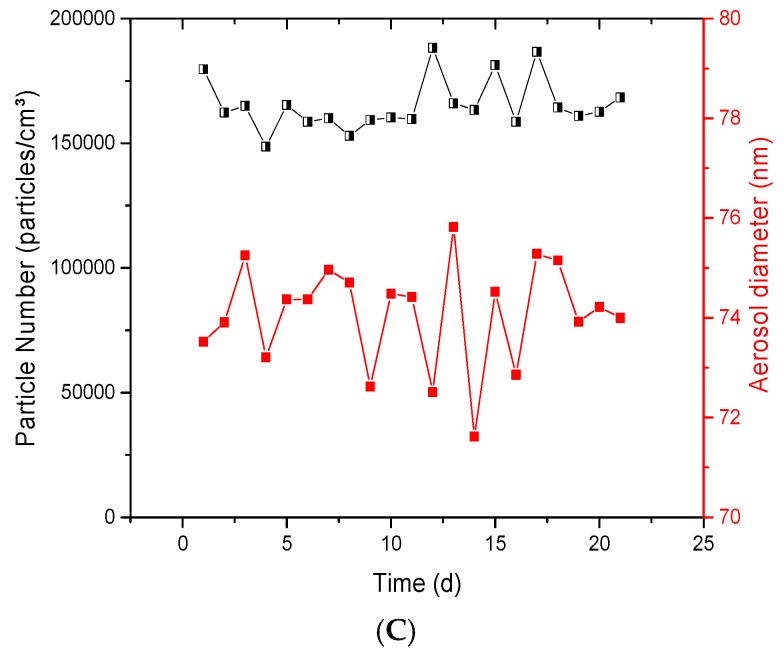
Evaluation of aerosols generated with the electrospray generator: (**A**) size distribution of typical TiO_2_ aerosols as function of mass concentration; (**B**) stability of aerosols over 6 h; (**C**) long-term stability of the number of particles and diameter in the aerosols over 21 days. The TiO_2_ (20 nm) suspension concentration of 100 mg/ml was used at a rate of 5, 25 and 35 μL/min in (**A**); 6, 12 and 25 μL/min in (**B**); 6 μL/min in (**C**) to generate an aerosol per day over 21 days. The red points represent diameters of particles in the aerosol (nm) monitored at specific time points over 21 days (see right ordinal axis).

For TiO_2_ aerosol generation with the electrospray method, concentrations reached were typically between 0.15 and 2.5 mg/m^3^ compatible with specifications of this generator, which has the ability to produce small aerosols with high particle numbers and small mass concentrations. Aerosol profiles were nanosized and the geometric mean diameters were between 73 and 76 nm (Figure 4A); the distribution was homogenous and unimodal with a *σ_g_* < 1.75. At 0.18 mg/m^3^, the number of particles was 188,200 particles/cm^3^ and the geometric mean diameter was 73.59 nm. The number of particles increased to 1,887,000 particles/cm^3^ at 1.6 mg/m^3^, while the diameter remained constant (73.34 nm; *σ_g_* of 1.73). For technical reasons, the electrospray was not able to generate stable aerosols over more than 6 h. Figure 4B shows the stability of these aerosols over the 6 h, with a variation of 8.46, 5.86 and 5.13% based on respective averages of 138,000, 239,000 and 510,000 particles/cm^3^. The repeated stability from day to day is depicted in Figure 4C. The average number and geometric mean diameter were stable, with a number of 165,000 ± 10,000 particles/cm^3^ and a geometric diameter of 74.1 ± 1.1 nm. All these results show that this generator has the ability to produce a good and stable dispersion of NPs in aerosols, applicable for exposure time shorter than 6 h, and for repeated day-to-day exposures.

### 3.2. Exposure Conditions Affecting the Aerosol Characteristics

**Figure 5:**
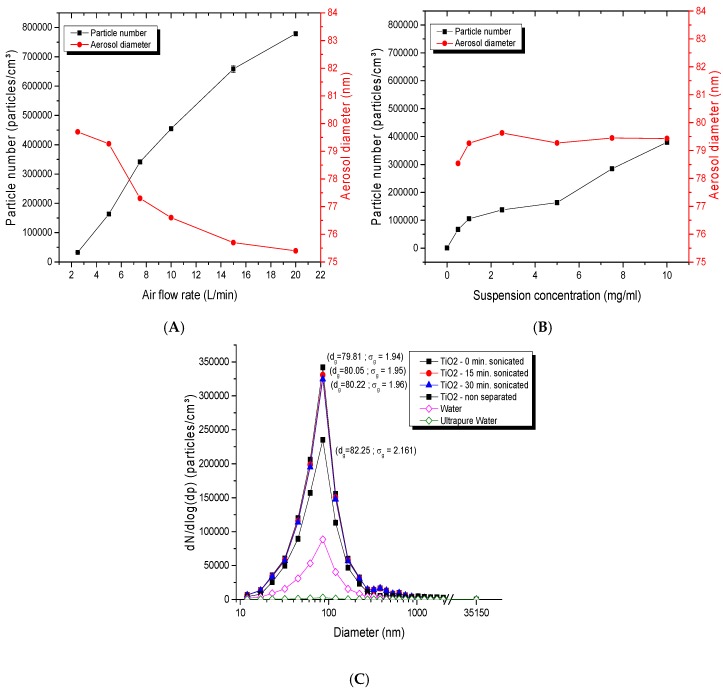
Parameters influencing collision-jet aerosol generation: average diameter and number of particles of TiO_2_ in the aerosols as function of: (**A**) airflow rate; (**B**) nanoparticle (NP) suspension concentration (with TiO_2_ (20 nm) suspension concentration in (**A**) and airflow rate in (**B**) at 5 mg/L and 5 L/min, respectively); (**C**) size distribution of TiO_2_ sonicated for different durations, use of deionized water compared to tap water, and effects of use of a laminar airflow separator (a suspension of 20 nm TiO_2_ NPs at 5 mg/mL, and ultrapure and unpurified water were used at an airflow rate of 5 L/min).

For the collision jet generator, varying the airflow rates between 2 and 20 L/min (Figure 5A) showed that the geometric mean diameter decreased with increasing airflow rates. For a suspension of 5 mg/mL and a generation rate of 2.5 L/min, the geometric diameter was 79.25 nm with a *σ_g_* of 1.93. This diameter decreased to 75.70 nm with a *σ_g_* of 1.93 when the flow rate was increased to 15 L/min. Conversely, the number of particles increased from 160,000 to 780,000 particles/cm^3^ with increasing airflow rates (from 2.5 to 15 L/min). On the other hand, Figure 5B showed that increasing NP suspension concentrations of TiO_2_ from 0.5 to 10 mg/mL did not have an impact on diameters, whereas the number of particles increased from 67,000 to 379,000 particles/cm^3^. The airflow rate and the NP suspension concentration thus appear to be two important parameters that can affect the mass concentration and number of particles in the exposure chamber; however, only the air flow rate affects the aerosol diameter. Moreover, Figure 5C shows that sonicating the NP suspension for 15 and 30 min (to disperse NPs before aerosolization in the collision jet) did not alter the aerosol characteristics such as the agglomeration state of the NPs in the aerosols. All the aerosols had low geometric standard deviations (*σ_g_* of 1.95 ± 0.01), a similar size (80 ± 0.2 nm), and the same number of particles and mass concentration (150,700 ± 4000 particles/cm^3^, 6.79 ± 0.02 mg/m^3^). Figure 5C also highlights that a non-filtered water presents a high background (39,300 particles/cm^3^) as compared to filtered water (1200 particles/cm^3^). Figure 5C further indicates that the use of a laminar separator led to a smaller aerosol size and reduced standard deviations (79.8 nm; *σ_g_* of 1.94) as compared to aerosols that did not pass through the separator (82.25 nm; *σ_g_* of 2.16).

**Figure 6:**
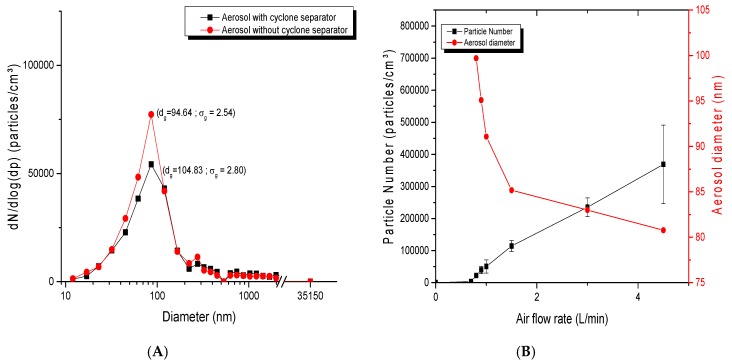
Parameters influencing dust jet aerosol generation: (**A**) size distribution of TiO_2_ aerosol with the use of a cyclone airflow separator. TiO_2_ nanopowders of 20 nm were used at an airflow rate of 5 L/min. ; (**B**) average diameter and number of particles of TiO_2_ (20 nm) in the aerosols generated as function of airflow rate.

For the dust-jet generator, as shown in Figure 6A, the number of particles increased with increasing airflow rates, while the particle diameter decreased. For an airflow rate of 0.8 L/min, the geometric mean diameter was 86.93 nm with a *σ_g_* of 2.31. This diameter slightly decreased to 80.77 nm with a *σ_g_* of 2.05 when the flow rate increased to 4.5 L/min. The airflow rate was thus an important parameter affecting the concentration, the number of particles but also the size of NP agglomerates in the aerosol. Figure 6B showed the impact of a cyclone separator on the separation of large agglomerates. It confirmed that the use of the cyclone separator generated finer and less dispersed aerosols (94.64 nm; *σ_g_* of 2.54), while its absence led to a higher particle size (104.83; *σ_g_* of 2.80), therefore showing that this device is effective in removing larger agglomerates.

**Figure 7:**
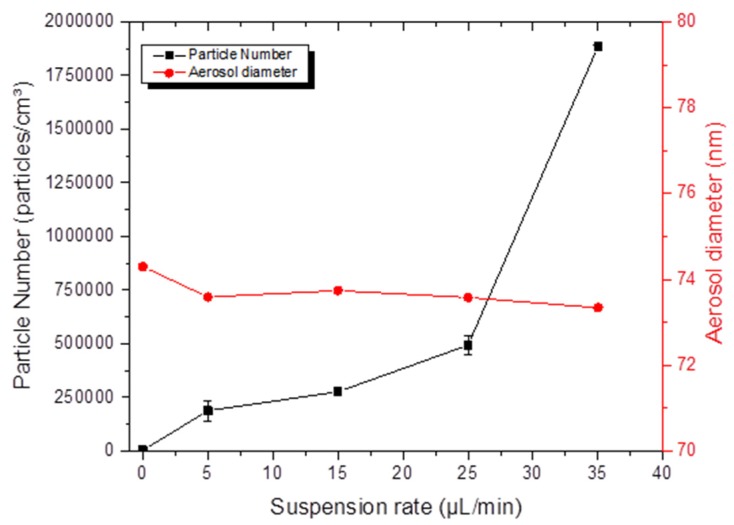
Parameters influencing electrospray aerosol generation: average diameter and number of particles of TiO_2_ (20 nm) in the aerosols as function of the NP suspension flow rate.

Finally, Figure 7 depicts the electrospray settings affecting the concentrations and particle diameters in the exposure chamber. The number of particles greatly increased with an increasing NP flow rate, while particle diameters remained constant. Thus, for a suspension flow rate of 5 and 25 μL/min, the geometric mean diameters were 73.34 and 73.58 mm with a *σ_g_* of 1.73, and the number of particles increased from 188,200 to 493,000 particles/cm^3^. Only the suspension flow rate was a malleable and important setting that can influence the NP number and the mass concentration.

### 3.3. Influence of the Initial NP Size on Aerosol Characteristics

**Figure 8:**
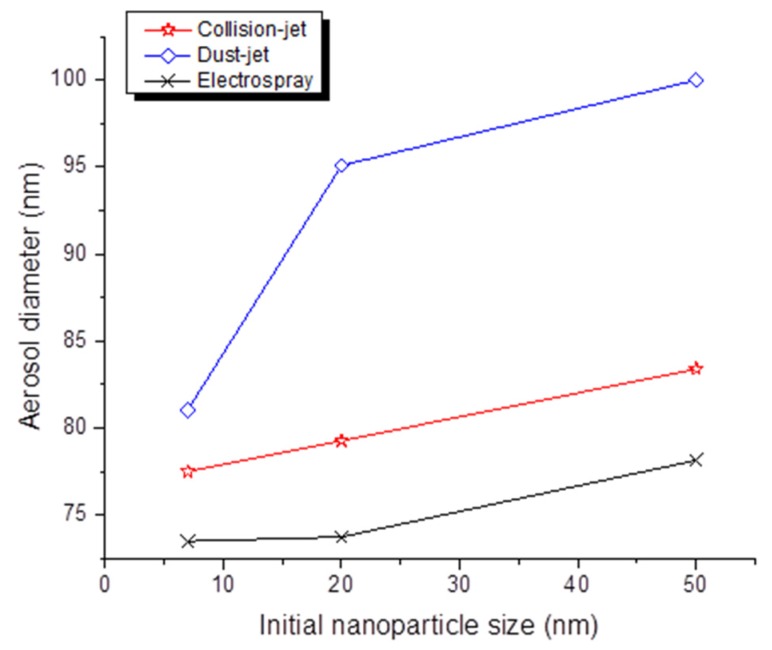
Average diameter of aerosols generated with the collision jet, dust jet and electrospray generators as function of initial NP size. For each NP size (7, 20 and 50 nm), aerosols were generated with the same number of particles, at 40,000, 160,000 and 250,000 particles/cm^3^ for the collision jet, dust jet and electrospray, respectively.

Using all three generators and considering a given concentration, Figure 8 shows that the initial size of the NPs influenced the geometric mean diameter of all aerosols, with rising diameters as initial NP size increased. For the collision jet, with a numeric concentration kept at ~160,000 particles/cm^3^, a mean diameter of 77.51, 79.27 and 83.39 nm was obtained for NP initial sizes of 7, 20 and 50 nm, respectively. The mean diameter was also higher with increasing initial NP size, ranging from 81.1 to 100 nm (~40,000 particles/cm^3^) when using the dust jet generator, and from 73.5 to 78.25 nm (~250,000 particles/cm^3^) using the electrospray generator.

### 3.4. Comparison of Aerosol Characteristics Depending on the Generator

Table 1 compares the overall characteristics and profiles of aerosols produced with the three generators. The dust jet generator produced the most highly mass concentrated aerosols (1.5–150 mg/m^3^) with large particle size (80 < *d_g_* < 100 nm). Conversely, the electrospray generator produced aerosols with low mass concentrations, but highly concentrated in particle number (200,000–1,800,000 particles/cm^3^) and with a smaller particle size (<75 nm). The collision jet was the most flexible generator capable of producing aerosols with low and high concentrations, and a size distribution of particles that remained variable between 75 and 80 nm.

### 3.5. TEM Characterization of NPs

**Figure 9:**
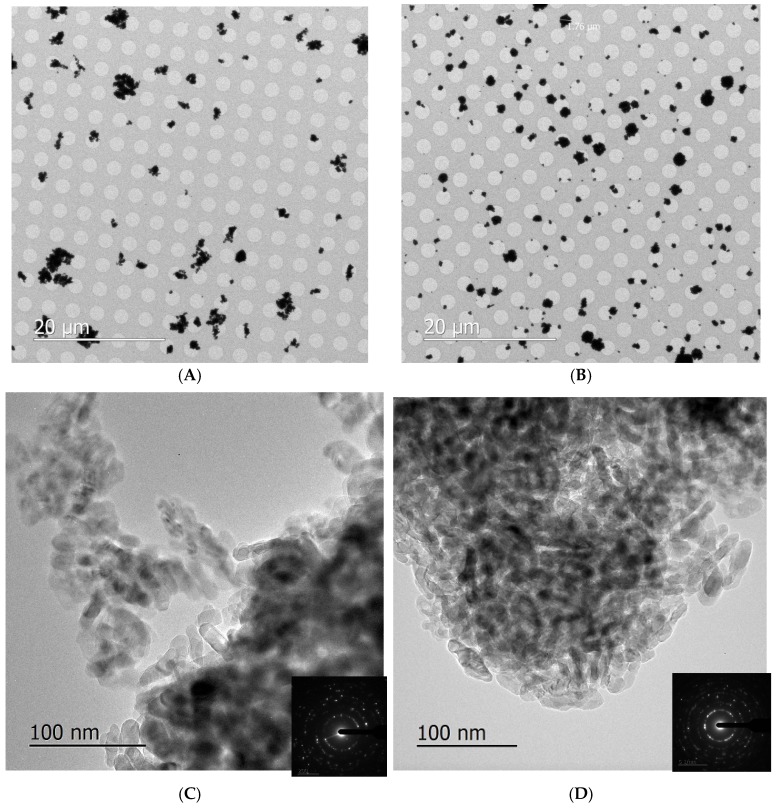
TEM images of NPs dispersed in the aerosol with the dust jet generator (**A**,**C**) and the collision jet generator (**B**,**D**) and collected on grids. The inset represents the corresponding selected area electron diffraction patterns confirming the nano-crystalline anatase TiO_2_ structure.

TEM investigation provided information on the morphology and the microstructure of the materials. Figure 9A,B presents two low-magnification micrographs showing the clusters of NPs collected on the TEM grids following aerosol generation with the dust jet and collision 6–jet. In general, NPs on the TEM grids were agglomerated into clusters with sizes between hundreds of nanometers and several microns; this high agglomeration of NPs on grids, as compared to what would be found in the aerosol, is expected, because of the natural behavior of NPs to cluster together. Therefore, it does not correspond to the agglomeration level of NPs breathed in by animals in the inhalation chamber. Figure 9C,D shows that the TiO_2_ NPs have the square prism shape typical of anatase; their sizes are between 10 and 30 nm, as expected. Anatase–TiO_2_ crystal structure is confirmed by the selected area electron diffractions with *a* = 3.78 Å and *b* = 9.51 Å. Diffraction rings around the central beam indicate that the grains are nano-crystals (see SAD in insets).

## 4. Discussion

For nanotoxicology studies, aerosols produced for inhalation exposures should have constant and uniform concentrations and be composed of particles smaller than 100 nm in diameter, which may be present as individual or as aggregates/agglomerates [21]. In this article, three generators have been evaluated and compared with the purpose of producing aerosols usable for such inhalation experiments. Evaluation of aerosols requires accurate and robust measurements by laboratory instruments, sufficiently reliable to provide continuous data records, with minimum intervention from the scientist who operates them [21]. It has been shown that nano-aerosols generated using nebulization techniques produce a size distribution composed essentially of smaller NP agglomerates, closer to toxicologically relevant mass concentrations and particle number [3,22,23,24]. In contrast, nano-aerosols that are produced by dry powder dispersion techniques generally consist of larger NP agglomerates, which are often encountered in the workplace [23]. In this study, parameters associated with the generation of small NP agglomerates (<100 nm) were described for the first time using dry and electrospray generation methods.

Our study showed the ability of the dust jet generator to produce a wide range of stable nano-aerosols, from 1.5 to 150 mg/m^3^, applicable for continuous 8 h and repeated 21-day animal exposures. This generation method, which eliminates the need for the addition of a solvent or a vehicle to aerosolize the NPs, is useful when evaluating TiO_2_ NP toxicokinetics or translocation from the lungs to extra-pulmonary compartments, effects on secondary target organs, as well as cytotoxic and oxidative stress responses. In their study, Schmoll et al. [17] reported having difficulties obtaining stable aerosols over short time periods, due to the compaction and adherence of dry nanopowders in the device. To produce stable and constant concentrations in our study, the nanopowders were unpacked and fluidized with a magnetic stirrer. This has led to a good dispersion of NPs in the device and the production of stable and constant aerosols over a prolonged period of time. Moreover, for all particles tested in our work, aerosols generated had homogenous size dispersions, but large geometrical standard deviations (2 < *σ_g_* < 3), and high geometrical diameters (80 < *d_g_* < 100 nm). These values remained smaller compared with those of other studies, where aerosols generated had mean sizes greater than 100 nm. Dust aerosolizers (airbrush generators) were also used by Rossi et al. [25], Ma-Hock et al. [26] and Schmoll et al. [17]. They reported the generation of TiO_2_ aerosols with high geometrical diameters: 100, 250 and 129 nm, respectively, from TiO_2_ nanopowders of 20, 25 and 21 nm. Other studies using dust aerosolizers reported high geometrical diameters of aerosols [27,28,29]. Our study has demonstrated the necessity to apply a sufficient force to separate or fragment nanopowders to form aerosols with a small size distribution. In the absence of solvent, NPs in powders are physically close and tend to agglomerate to form larger particles. Indeed, our results show that particle size decreased (from 100 nm to 80 nm) as the generator airflow rate was increased (from 0.7 to 4.5 L/min), due to the fragmentation of larger particles. The fragmentation and separation of large particles in aerosols were also achieved using an airflow dilution at high velocity and the use of a cyclone separator.

As for the collision jet generator, it allowed the generation of a wide range of size and mass concentrations. Aerosols generated were homogenous and uniform, with small geometrical mean diameters between 75 and 85 nm, and small geometrical standard deviations (*σ_g_* <2) for all tested particles. The suspension of NPs in a liquid vehicle with stirring and the use of a high airflow rate into the generator produced finer and homogeneous aerosols, applicable for continuous 8-h inhalation and repeated day-to-day exposure (up to 21 days). Based on the basic droplet formation, as airflow velocity was increased, greater impaction of the NP suspension against the glass vessel was observed, producing more droplets, and in turn the formation of smaller particles [30]. Moreover, the use of highly concentrated NP suspensions in the generator allowed for the production of more concentrated aerosols, while the diameter remained constant. Schmoll et al. [17] also tested the collision jet with 21 nm TiO_2_ NPs. Their set up was able to produce a constant aerosol over 30 min with a small geometrical standard deviation of 1.62, but with a high geometrical mean diameter of 151.2 nm. Grassian et al. [31] also reported the generation of aerosols with the collision jet using 21 nm TiO_2_ NPs, but again high geometrical mean diameter of 138.8 nm was obtained with a standard deviation of 1.44. In the study of Noël et al. [24], the collision jet was also used to generate aerosols using 5 nm TiO_2_ NPs. This device was placed in parallel with a Delavan siphon spray nebulizer that uses very high airflow to reduce the size of the droplets, which allowed for the lowering of the overall median diameter (31 nm) of the aerosol. In our study, we demonstrated that high airflow rates in the generator combined with airflow dilution and a laminar separator are necessary to produce small aerosols below 100 nm. Unexpectedly, the dispersion of NP suspensions by sonication, prior to aerosol generation, did not alter the aerosol size or homogeneity. On the other hand, the importance of using deionized water to generate the NP suspension, obtain a homogenous distribution, and avoid contamination peaks, has been confirmed in our study, in line with the results of Schmoll et al. [17].

With regard to electrospray aerosolization, many generators have been described as being able to produce small aerosols close to initial NP size [19], but none of these generators were used to expose to animals. The electrospray used in our study had the specificity of generating nano-aerosols from a liquid suspension with a balance of electric charges. For all particles tested with the electrospray generator, it was demonstrated that our device generated uniform aerosols with a small geometric particle diameters (between 74 and 77 nm) and small geometrical standard deviations (<1.8), but the initial size of particles could not be retained once aerosolized. Different results were obtained by Suh et al. [14], where initial size of NPs (5, 10 and 20 nm) were maintained in the aerosols. This difference of size between initial NPs and particles in the aerosols in our study can be explained by the use of NPs without a colloidal dispersion, which can form agglomerates in suspension. Agglomerates may be colliding in the aerosols and contribute to increase mean size. This hypothesis is supported by results of Schmoll et al. [17] with an electrospray generator, showing that the use of non-colloidal suspensions of TiO_2_ NPs generated aerosols with larger diameters (on average 24 nm) than colloidal suspension (on average 5 nm). In the current study, it was also shown that the electrospray offers uniform and homogenous aerosol concentrations during 6 h or less. After 6 h, the condensation of water in the reaction chamber increases, and this causes electric short circuits stopping the generation. However, our study showed that electrospray generation is stable up to 6 h, and may be applicable for repeated day-to-day exposures (21 days).

Overall, performance comparison of the three generation methods based on criteria needed for animal exposures, namely the choice of NPs, particle size distribution, mass concentration, particle number and exposure duration, highlighted that the dust jet was the most straightforward to use. It easily aerosolizes NPs from its powder form, which is the most available form. The collision jet and electrospray generators require the suspension of NPs in water or in a biological buffer.

Regarding the particle size distribution, few studies were able to produce aerosols with a size distribution under 100 nm, as was achieved in the present study with the generators used. Most studies produced aerosols with median particle size between 100 and 250 nm. The electrospray produced the finest aerosols (on average 70–75 nm), but it was not possible to change the size of the aerosols produced. The collision jet also generated small aerosols, but with a slightly higher average size than the electrospray (75–80 nm); it was, however, possible to modify the aerosol diameters by adjusting the airflow rate. Although the dust jet was easy to use, it produced larger average-size aerosols (>80 nm) but like the electrospray, the airflow rate could be adjusted to set the diameters to values up to *circa* 100 nm.

Depending on the type of study, it is necessary to choose the generator that produces the sought concentrations. The three generators produced different concentration ranges. For highly concentrated aerosols, the dust jet was the most appropriate generator. It easily produced stable aerosol concentrations at 15 mg/m^3^, although it was unable to produce aerosols below to 1.5 mg/m^3^ or aerosols with high particle numbers. This generator may be more adapted for exposure to high concentrations during short time periods, rather than long or repeated exposures to low concentrations. The most versatile generator tested was the collision jet; it easily produced aerosols at both low (0.500 mg/m^3^, 30,000 particles/cm^3^) and high concentrations (30 mg/m^3^, 800,000 particles/cm^3^), stable for hours. For very low concentration aerosols, the electrospray was the best generator; it was able to produce aerosols between 0.150 and 2.5 mg/m^3^ with a high number of particles.

All three generators were shown to produce stable aerosols for acute and repeated exposures. For 8 consecutive hours of generation with the dust jet and collision jet, variations in mass concentrations were <10% at low, mid and high concentrations. The electrospray was also able to produce stable aerosols with low variations, below 10%, but for a maximum of 6 h. For repeated exposures over several consecutive days, needed for sub-acute evaluation, it was demonstrated that the three generators were able to generate stable aerosols, with small day-to-day variations in geometric diameters and concentrations.

## 5. Conclusions

The three generators used thus appeared suited for nanoparticle inhalation toxicology studies. Methods were stable and applicable for continuous exposures in animals over hours and repeated over several days. The three generators were capable of producing small aerosol size distribution with geometric means below 100 nm. Other conventional systems usually produce aerosols with a size between 100 and 250 nm. Our results were mainly due to an optimized use of these generators to aerosolize NPs, like high velocity and pressure airflow or particle separation compartments. However, the nebulizer collision jet aerosolizer was the most versatile generator, producing both low and relatively high concentrations. The inhalation system for experimental studies in animals will be useful to assess the impact of conditions such as the exposure concentration, numerical counts and agglomeration state on the toxicokinetics and toxicity of various NP aerosols.

## Figures and Tables

**Table 1:** Comparison of size distribution characteristics with the three aerosol generators tested.

Generator	Mass Concentration (mg/m^3^)	Particle Number (particles/cm^3^)	Geometric Mean (nm)	Geometric Standard Deviation	Daily Stability (h)	Repeated Daily Exposure (d)
Dust Jet	1.5–150	10,000–400,000	80–100	*σ_g_* < 3	8	21
Collision Jet	0.5–30	30,000–800,000	75–80	*σ_g_* < 2	8	21
Electrospray	0.15–2.5	200,000– >1,800,000	70–75	*σ_g_* < 1.8	6	21
